# Insights from molecular dynamics and DFT calculations into the interaction of 1,4-benzodiazepines with 2-hydroxypropyl-βCD in a theoretical study

**DOI:** 10.1038/s41598-023-36385-w

**Published:** 2023-06-18

**Authors:** Mokhtar Ganjali Koli, Rahime Eshaghi Malekshah, Hossein Hajiabadi

**Affiliations:** 1 InSilicoSci Computational Research Centre, Nikopardazesh Ltd., Karaj, Iran; 2 Department of Chemistry, University of Kurdistan, Sanandaj, Iran; 3 Department of Chemistry, Faculty of Science, Semnan University, Semnan, Iran

**Keywords:** Physical chemistry, Theoretical chemistry, Computational chemistry, Drug delivery

## Abstract

This study delves into the interaction between benzodiazepine (BZD) drugs and 2-hydroxypropyl-β-cyclodextrin (2HPβCD), a cyclodextrin (CD) known to improve drug delivery and enhance therapeutic outcomes. We find that the 2HPβCD’s atoms become more rigid in the presence of chlordiazepoxide (CDP), clonazepam (CLZ), and diazepam (DZM), whereas they become more flexible in the presence of nordazepam (NDM) and nitrazepam (NZP). We also investigated the structure of 2HPβCD and found that loading these drugs increases both the area and volume of the 2HPβCD cavity, making it more suitable for drug delivery. Moreover, this research found that all drugs exhibited negative values for the binding free energy, indicating thermodynamic favorability and improved solubility. The binding free energy order of the BZDs was consistent in both molecular dynamics and Monte Carlo methods, with CDP and DZM having the highest affinity for binding. We also analyzed the contribution of different interaction energies in binding between the carrier and the drugs and found that Van der Waals energy is the primary component. Our results indicate that the number of hydrogen bonds between 2HPβCD/water slightly decreases in the presence of BZDs, but the hydrogen bond’s quality remains constant.

## Introduction

Benzodiazepines (BZDs) are a class of widely prescribed psychoactive drugs that have been used to treat a variety of conditions since the 1960s, such as anxiety, insomnia, and seizures^1^. BZDs work by enhancing the activity of the neurotransmitter gamma-aminobutyric acid (GABA) in the brain, resulting in sedative, anxiolytic, and muscle relaxant effects^2^. However, despite their therapeutic benefits, BZDs have a high potential for abuse, dependence, and withdrawal, making their long-term use controversial^3^. One approach to address these issues is to improve the delivery and bioavailability of BZDs by using drug delivery systems such as cyclodextrins (CDs). CDs are cyclic oligosaccharides that have a hydrophobic cavity and a hydrophilic exterior, allowing them to form inclusion complexes with various guest molecules, including drugs. The resulting CD-drug complexes can improve drug solubility, stability, and absorption, leading to enhanced therapeutic efficacy and reduced adverse effects^4^. Since now, several studies have investigated the complex formation of BZDs with CDs and their potential as drug delivery systems. They proved that CDs such as β-CD, hydroxypropyl-β-cyclodextrin (HPβCD), and sulfobutylether-β-cyclodextrin (SBEβCD) can improve the solubility and bioavailability of BZDs such as diazepam, lorazepam, and clonazepam^5–7^. In addition, CDs are being used to mask the bitter taste of BZDs, making them more palatable and improving patient compliance^8^. One advantage of CD-based drug delivery systems is their ability to target specific sites in the body, including the central nervous system (CNS)^9^, where BZDs exert their pharmacological effects. CDs can cross the blood–brain barrier (BBB), a selective barrier that protects the brain from potentially harmful substances, by interacting with specific transporters or by modulating the BBB permeability. This allows CDs to deliver BZDs to the brain, increasing their therapeutic efficacy while reducing their peripheral side effects^10,11^. Another advantage of CD-based drug delivery systems is their potential to reduce the toxicity and adverse effects of drugs. Complexation of drugs with CDs, for instance, can reduce their binding to plasma proteins, causing to drug accumulation and toxicity^12,13^. CDs can also reduce the metabolism and elimination of BZDs by inhibiting the activity of enzymes such as cytochrome P450, leading to a longer half-life and sustained drug action^14,15^. This can reduce the possibility of withdrawal symptoms and rebound effects associated with BZDs. In addition, CDs can modulate the release of BZDs, enabling controlled drug delivery and sustained drug action^16^. This can be achieved by using different CDs, modifying their structure, or by incorporating them into drug delivery systems such as nanoparticles or liposomes. This can improve the pharmacokinetics and pharmacodynamics of BZDs, leading to a more predictable and effective therapeutic response^17^. One of the most powerful methods for predicting the properties of CDs as well as the other field of studies is molecular dynamics simulation, which enables the reproduction of a wide spectrum of experimental properties^18–21^. Over the past few years, notable progress has been achieved in the domain of environmental pollutant eradication, particularly in the realm of eliminating substances such as carbon dioxide gas. These advancements could be found to the incorporation of diverse methodologies, including molecular dynamics simulations and quantum advancements, alongside the utilization of membranes and organic-metal frameworks^22–26^. In this study, we used molecular dynamics (MD) simulations and density functional theory (DFT) calculations to provide a detailed insight into the thermodynamics and kinetics of the BZD-CD complexes. To provide insight into the mechanisms that govern their interaction^27,28^ and paving the road for the development of more effective BZD-CD drug delivery systems, we investigated a wide range of their properties, including binding affinity, orientation, dynamics of the drug molecules inside the CD cavity, conformational change, and the fluctuation of the CD molecule. The main purpose of this research was to examine the mutual effects of CD-BZD interactions and to achieve a thorough understanding of the dynamics and thermodynamics of the formation of host–guest systems composed of a 2-Hydroxypropyl-βCD (2HPβCD) molecule and various 1,4-Benzodiazepines derivatives such as Diazepam (DZM), Chlordiazepoxide (CDP), Clonazepam (CLZ), Nitrazepam (NZP), and Nordazepam (NDM).

## Computational details

### Molecular dynamics simulations

**Figure 1:**
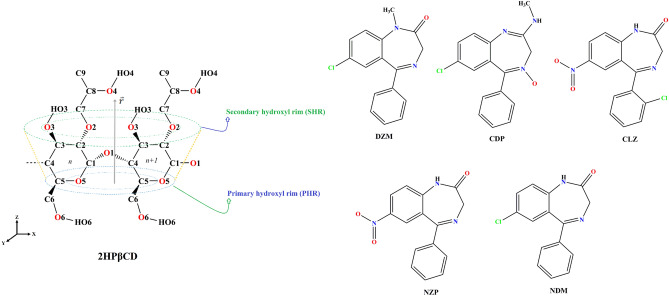
Molecular structure of 1,4-benzodiazepines derivatives and 2-hydroxypropyl-βCD.

Molecular structure of 1,4-Benzodiazepines (BZDs)^29^ and 2HPβCD^30–32^ are illustrated in Figs. 1 and S1. Thirteen distinct systems were designed and simulated. The first system, serving as a reference, consisted of only one 2HPβCD molecule and water molecules. The next six systems each comprised a single drug and water molecules. The remaining six systems each contained one drug molecule alongside the 2HPβCD and water molecules. All molecular dynamics (MD) simulations were conducted in the NPT ensemble utilizing the GROMACS 2020 package^33,34^. The neutral form of the BZD molecules and 2HPβCD was used by the GROMOS 54a7 force field^35,36^, which has been validated for its structural parameters^27^.

**Figure 2:**
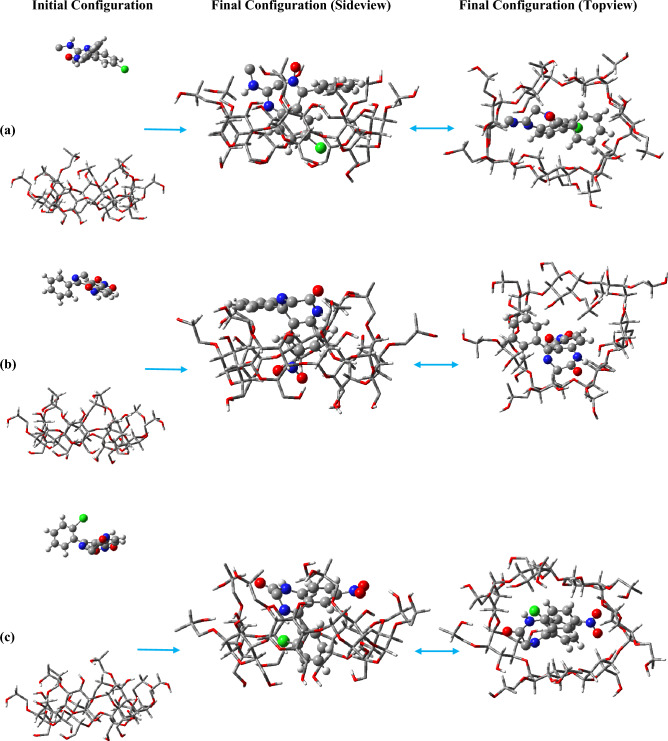

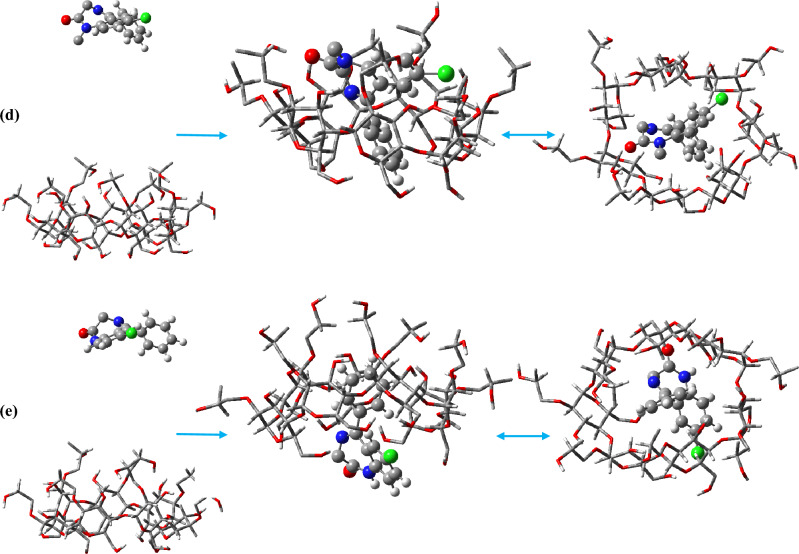
Snapshots of the initial and final configurations of CDP (**a**), NZP (**b**), CLZ (**c**), DZM (**d**), and NDM (**e**) toward 2HPβCD.

To prevent any imposing interaction between the drug and the 2HPβCD molecules, the initial configurations were set such that the BZDs are about 1.5 nm away from the secondary hydroxyl rim (SHR), as shown in Fig. 2.

All systems were hydrated with 4000 water molecules, and periodic boundary conditions were applied in all three dimensions. For water molecules, the extended simple point charge model (SPC/E) was used^37^. Undesirable atomic contacts were removed by performing a steepest descent energy minimization^38^, and the systems were equilibrated in the NVT and NPT ensembles for 1 ns and 9 ns, respectively. After equilibration, the simulations were run for 100 ns while keeping the bond lengths constrained by the LINCS algorithm^39^. The temperature was maintained at 300 K using the V-rescale coupling method^40^ with a time constant of 0.1 ps, and the pressure was held constant at 1 bar utilizing the Parrinello-Rahman barostat, with coupling time constant 2 ps^41^. The leap-frog algorithm with a time step of 2 fs was used to integrate Newton's equations of motion^42^. The particle mesh Ewald (PME)^43^ technique was utilized to consider the long-range Coulomb interaction, and a cut-off distance of 1.2 nm was applied for both Coulomb and Van der Waals (vdW) interactions. The drug concentration in all drug-containing systems was ~ 13.5 mM. In this research, GaussView 5.0 software was employed to visualize the geometries^44^, while two-dimensional (2D) chemical structures were created using ChemDraw Professional 16.

### Free energy computations

The Bennett acceptance ratio method (BAR)^45^ was used to calculate the free energy differences between Hamiltonians at different values of λ. A thermodynamic cycle based on alchemical free energy calculations^46^ was employed to determine the binding free energy. The computation of the free energy resulting from the induction between the drug and its environment involved slowly turning off the potential energy of interaction between the drug molecules and their environment using a λ parameter that ranges from 0 to 1. To calculate the ΔG_solv_, 21 λ points were used. Coulombic solute–solvent interactions were turned off with larger λ at the beginning compared to vdW terms^47^ to prevent unstable Coulombic interactions that can cause unstable configurations and unreliable energies. We used an evenly spaced Δλ = 0.05 from 0 to 1 for vdW interactions, while Coulombic interactions started with Δλ = 0.1 for decoupling. Soft-core potentials with α = 0.5, σ = 0.3, and p = 1 were employed to prevent the overlapping of solute–solvent atoms for low λ values. The final configuration of each simulated system was used as the initial computing file for free energy calculations. Each simulation was 5 ns long, and the last 1 ns was used for extracting the free energy.

### DFT calculations

To assess the ability of 2HPβCD to carry benzodiazepine-based drugs, we applied density functional theory (DFT) calculations. First, we used Material Studio 2017 to draw all the structures, DZM, CDP, CLZ, NZP, NDM, and 2HPβCD. Then, all structures were optimized using the DMOL3 module^48^, the density functional approach (DFT-D)^49^, and the GAA using the Perdew-Burke-Ernzerhof (PBE) functional^50^ to investigate the electronic structure of atoms/molecules, energy levels, and the frontier orbitals. A Pople’s split-valence double-zeta basis set, 6–31 G, was chosen for all the DFT calculations because of its high convergent ability. The adsorption locator module was applied based on Forcefield; universal in Material Studio 2017^25,51^.

### Ethics declarations

NikouPardazesh Learning Center provided financial assistance, such as paying for salaries, equipment, supplies, and other expenses, for the research project related to developing the idea, designing the study, gathering data, analyzing results, deciding to publish, or preparing the manuscript, as indicated in Document No. 28.

## Results and discussion

### Dynamic and structural properties

#### Distance and mechanism of loading

**Figure 3:**
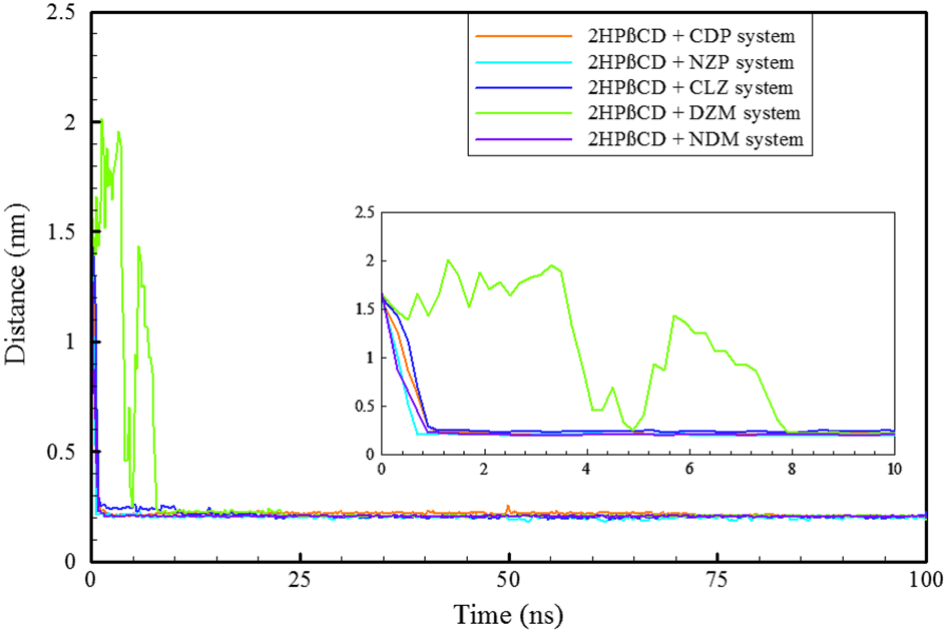
Time evaluation the distance between BZDs and the center of 2HPβCD.

The mechanism of drug-carrier interaction is a significant field of study in pharmaceutics. Understanding the mechanism can help improve drug delivery and enhance therapeutic outcomes, and leads to better patient outcomes^27,52,53^. To examine the mechanism, we investigated the center of mass (COM) motion of the BZD molecules relative to the 2HPβCD cavity center, as shown in Fig. 3.

The results revealed that all BZD molecules quickly bound to the 2HPβCD cavity, except for DZM, which takes longer to enter. DZM enters the cavity after approximately 8 ns. The molecules had the chance to interact with the cavity from either direction because their initial positions of 2HPβCD were further away from the cutoff distance. In contrast to the other four molecules, which enter from the secondary hydroxyl rim, NDM enters from the primary hydroxyl rim (PHR) and interacts with the 2HPβCD cavity, Fig. 2. Moreover, NDM enters the cavity implicitly through the benzene ring side (which bears the numbers 1′, 2′, etc., Fig. S1), whereas their adjacent rings—which are composed of nitrogen and chlorine—are located on the cavity's exterior, close to the water. This finding supports the idea that BZDs interactions with 2HPβCD can cause in various BZDs conformers and flexibility, which we will discuss further. CDs can protect drugs from degradation by forming inclusion complexes that shield them from light, oxygen, and other degradative factors^54,55^. As a result, it appears that 2HPβCD provides greater protection for the CDP, NZP, CLZ, and DZM molecules because they are fully contained within the cavity of 2HPβCD, thereby remaining shielded from the influences of the chemical environment. The host–guest systems were formed in a 1:1 ratio, it seems that in order to provide better protection for NDM, higher concentrations of 2HPβCD such as 2:1 ratio may be required.

#### Root mean square fluctuation (RMSF)

**Figure 4:**
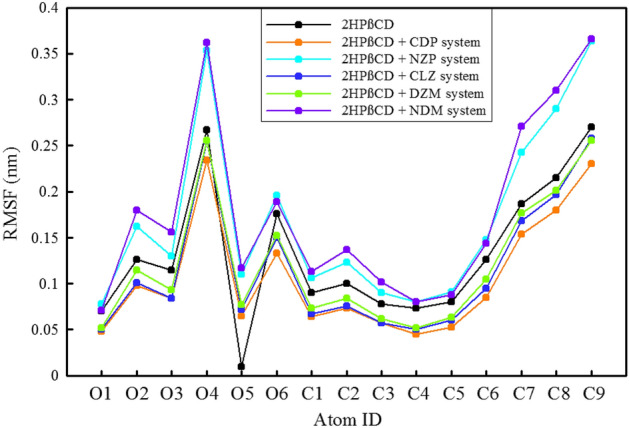
Root mean square fluctuation (RMSF) of heavy atoms of 2HPβCD in presence of different simulated system.

The RMSF values can reveal regions of the molecule that are highly flexible and undergo large conformational changes, as well as regions that are more rigid and less likely to move^56,57^. BZDs are rigid in their interaction with 2HPβCD, as demonstrated by the small RMSF changes of less than 1 Å when examining the flexibility of these drugs, Figs. S2, S3. However, NDM is an exception. Atoms 14 and 28 of NDM (corresponding to carbon and chlorine atoms, respectively) as well as atoms 17 and 19 (to a lesser extent) exhibit significant alterations. The RMSF changes are most noticeable in the region that is outside the 2HPβCD cavity, where the NDM atoms come into contact with water molecules. Simultaneously, the flexibility of 2HPβCD atoms was also investigated, Fig. 4.

The results show that the O4 and O6 are highly flexible because they are fully exposed to water. The O2 is less flexible due to its functionalization and added hydroxypropyl heavy groups, but it is still available to water molecules. The O3 is more exposed to the solvent atoms, due to hydroxypropyl groups, and has formed more hydrogen bonds that cause stability and less flexibility in the O3 atoms (as discussed in “Hydrogen bonds”). The O1 and O5 are located inside the molecule's main skeleton and ring structure, which restricts their flexibility. O5 experiences drastic changes in the presence of NDM due to their loading mechanism, which is from the primary hydroxyl rim. In the NZP-containing system, NZP entry from the side of the bulky nitrite group into the cavity causes repulsion with O5, resulting in a significant increase in its flexibility. The carbon atoms within the ring of 2HPβCD (specifically, C1 to C5) exhibit limited flexibility. On the other hand, C6 to C9 are more flexible due to having more degrees of freedom and not being restrained by other atoms in the 2HPβCD structure. Consequently, the presence of CDP, CLZ, and DZM causes the 2HPβCD atoms to become less flexible and more rigid. Conversely, the presence of NDM and NZP leads to an increase in the flexibility of 2HPβCD atoms.

#### Structural properties

CDs have unique structural features that account for their wide variety of applications^58^. We investigated a set of the most important 2HPβCD structural changes in different simulated systems, summarized in Table 1. An explanation of how to compute both the area and volume of the cavity was provided on SI (page 6) and these approaches have been well confirmed in other simulation studies^59,60^. In the reference system, A_PHR_ (1.22 nm^2^) is slightly larger than the A_SHR_ (1.15 nm^2^), while with native βCD, it is proven that A_SHR_ is larger than A_PHR_^59,61^. This study shows that functionalization (specifically, hydroxypropylation at the O2 position) of βCD causes a change in this balance. As a result, in 2HPβCD compared to βCD, the main skeleton becomes more cylindrical while A_MID_ (related to the O1 atoms ring) remains the same. In addition, A_PHR_ increases and A_SHR_ decreases. However, a complete cylinder cannot form because there is still a discernible difference between the surface areas in 2HPβCD. With the simultaneous increase of A_SHR_ and A_MID_, the tendency to adopt a cylindrical shape is greatly enhanced by the entry of CDP, DZM, and CLZ. This can be explained by the loading mechanisms of these drugs from the SHR side that cause an increase in A_SHR_. In contrast, NDM binds from the PHR side, leading to a noticeable increase of A_PHR_ (and to a lesser extent of A_MID_), and the presence of these drugs causes 2HPβCD to adopt a truncated cone shape. NZP, which enters into the cavity from the bulky atoms side and is placed close to hydroxyl groups of PHR, can undergo a significant reduction in A_PHR_. This is due to the attractive interaction between the seven hydrogen atoms of the hydroxyl group and the two oxygen atoms of NZP. Because of the lack of flexibility of the O1 atoms (discussed in "Root mean square fluctuation (RMSF)" section), the ratio of distances between the O1 atoms is relatively unchanged, and the circularity of this ring (Ω_O1_) remains intact in all simulated systems. However, in BZD-containing systems, the circularity of Ω_O2_ and Ω_O6_ increases, with this effect being more significant for SHR. The results align with the observations regarding the flexibility linked to every group of atoms. The distances between the middle and the primary rings (*h*_12_) and the middle and the secondary rings (*h*_16_) provide valuable information about 2HPβCD. For the native βCD, *h*_12_ and *h*_16_ are 0.24 and 0.31 nm, respectively^60^. Table 1 demonstrates that in the reference system, the volume of the cavity (*V*_C_) was significantly reduced by shortening the distances between the rings, particularly *h*_12_.

**Table 1:** Structural parameters describing molecular arrangement of 2HPβCD in different simulated systems.

Systems	Parameter								
	*A*_PHR_ (nm^2^)	*A*_MID_ (nm^2^)	*A*_SHR_ (nm^2^)	Ω_O1_	Ω_O2_	Ω_O6_	*h*_12_ (nm)	*h*_16_ (nm)	*V*_C_ (nm^3^)
Water + 2HβCD (reference)	1.22	0.79	1.15	0.97	0.83	0.63	0.14	0.23	0.35
Water + 2HβCD + DZM	1.27	0.83	1.25	0.96	0.96	0.70	0.21	0.31	0.53
Water + 2HβCD + NDM	1.43	0.82	1.11	0.98	0.96	0.90	0.22	0.27	0.51
Water + 2HβCD + CDP	1.24	0.86	1.27	0.96	0.96	0.90	0.23	0.32	0.57
Water + 2HβCD + NZP	1.17	0.82	1.25	0.97	0.92	0.78	0.20	0.29	0.48
Water + 2HβCD + CLZ	1.29	0.84	1.25	0.97	0.98	0.66	0.21	0.30	0.54

*A*_PHR_, *A*_MID_, and *A*_SHR_ are the cavity areas of primary hydroxyl rim, middle rim, an secondary hydroxyl rim (the rings that are formed by connecting the O6, O1, and O3 atoms, respectively); *h*_1j_ is the distance between the center of mass of O1 atoms and the center of mass of Oj atoms; Ω_Xi_ is the circularity of rim comprising Oi atoms defined as the ratio of the smallest to the largest distance between any pair of Oi atoms in the rim; 2HPβCD height, *h*, is the distance between the centers of mass of the primary and the secondary hydroxyl rims (*h* = *h*_12_ + *h*_16_); Vc is the volume of cavity for 2HPβCD in different simulated systems.

However, given that DMZ, CDP, NZP, and CLZ are completely loaded into the 2HPβCD cavity and have increased the *V*_C_ (Fig. 1), it seems that this is not an obstacle to drug loading. Although NDM only occupied the cavity partially, it caused a substantial increase in *V*_C_. The NZP-containing system showed a minimal expansion in *V*_C_. This phenomenon can be explained by the formation of hydrogen bonds, which occur between NZP/O1 and O2 atoms at the same time (as discussed in the “Thermodynamic properties” section). This interaction acts as a connecting element between the two and inhibits *V*_C_ increase.

#### Relative shape anisotropy

Relative shape anisotropy (*κ*^2^ or RSA) in polymers refers to the degree to which the shape of a polymer molecule deviates from a perfect sphere or a perfect cylinder^62,63^. RSA is an important parameter for drug delivery as it affects the distribution and retention of drug carriers in the body. This parameter influences the transport and diffusion of drug carriers through biological barriers, such as cell membranes and tissue extracellular matrix. Understanding the RSA of drug carriers can improve drug delivery efficacy and reduce potential side effects^64,65^. The principal moments of the radius of gyration (Rg) are frequently arranged in the order λ_1_ ≥ λ_2_ ≥ λ_3_, and from the sum of these principal moments, the square of the Rg is obtained so that: ${\mathrm{R}}_{\mathrm{g}}^{2}={\uplambda }_{1}+{\uplambda }_{2}+ {\uplambda }_{3}$. Then, with the principal moments, *κ*^2^ could be achieved as follows^66^:

1 $${\kappa }^{2}=1-3\frac{({\lambda }_{1}{\lambda }_{2}+{\lambda }_{2}{\lambda }_{3}+{\lambda }_{1}{\lambda }_{3})}{{({\lambda }_{1}+{\lambda }_{2}+{\lambda }_{3})}^{2}}$$

**Figure 5:**
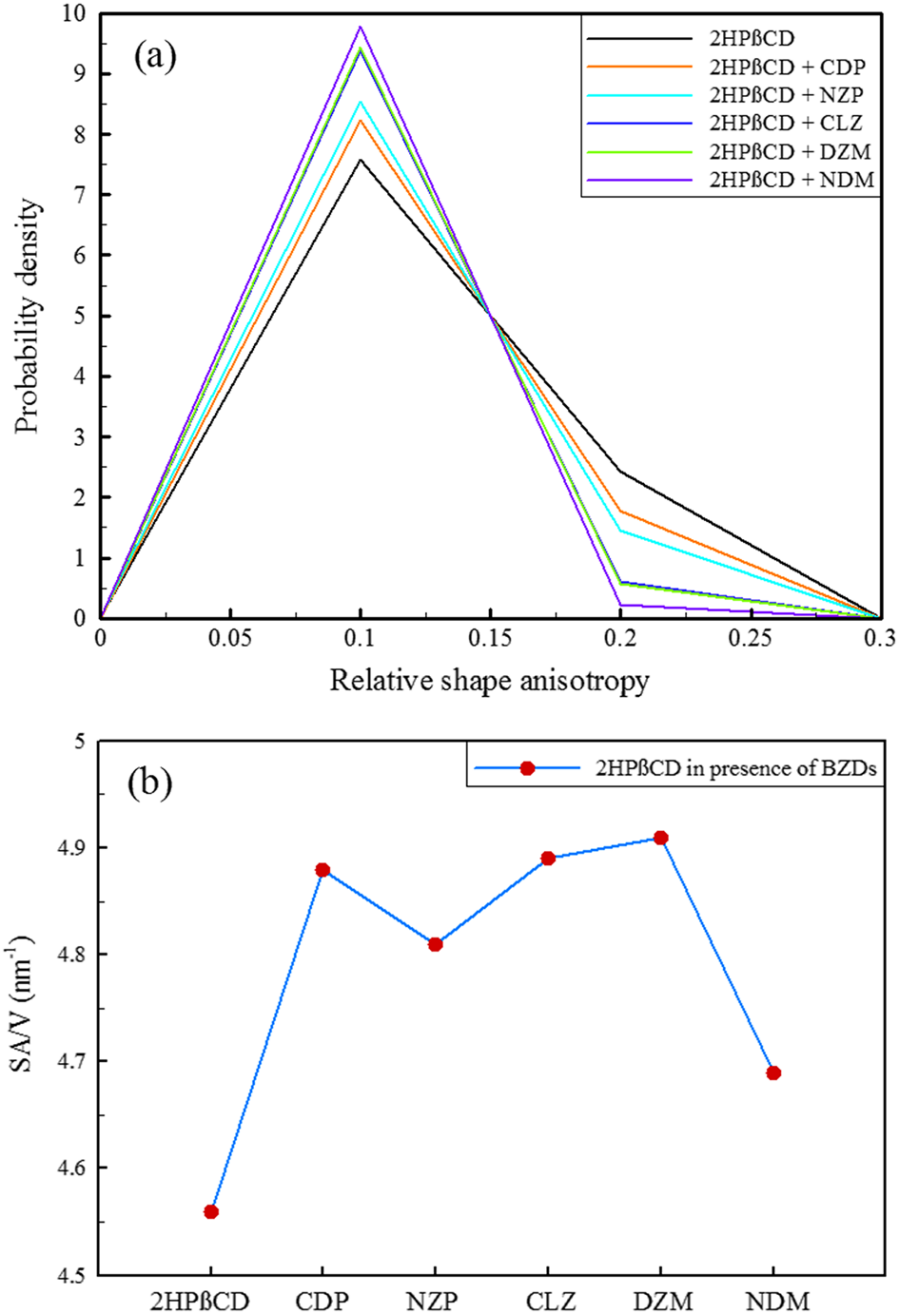
Comparison of the distributions of the relative shape anisotropy (**a**), and the surface-to-volume ratio (**b**) in different simulated systems.

*κ*^*2*^ can take values between 0 and 1, where a value of 0 corresponds to a highly symmetric conformation of a polymer, and it reaches 1 for an ideal linear chain. The relative shape anisotropy of 2HPβCD in the presence and absence of BZDs was examined. The probability density for the reference system (2HPβCD/water) was significant in the range of 0.2 (less spherical) and 0.1 (more spherical), as shown in Fig. 5a. The results indicated that when drugs were loaded inside the 2HPβCD cavity, the probability density decreased sharply at 0.2 and increased around 0.1 for all the BZD-containing systems. This suggests that the 2HPβCD structure became more spherical in the presence of the drugs. The presence of NDM caused an even greater increase in sphericity, while DZM and CLZ had similar effects on 2HPβCD, resulting in a complete overlap of their lines in Fig. 5a.

The observations indicate that the elongation of the object along its principal axes was closer in magnitude and direction in different directions, leading to a more spherical structure of 2HPβCD in drug-containing systems. Even though the more spherical structure of 2HPβCD could be considered a favorable dynamic factor for dissolution and drug delivery, there is more to it. The increased surface area of nanoparticles compared to their volume allows for greater interaction with their environment, making them useful for a wide range of applications, such as drug delivery and catalysis^67,68^. Therefore, we evaluated the ratio of the solvent-accessible surface area (SASA) of the 2HPβCD to its volume, Fig. 5b. As can be seen, when all BZDs are present, the SA/V ratio of 2HPβCD increases. The highest increment corresponds to DZM, followed by CLZ, CDP, and NZP. The presence of NDM, however, resulted in only minor changes, which may be due to its interaction mechanism and inability to fully enter the 2HPβCD cavity. As a result, they have fewer interactions with the 2HPβCD atoms, and their surface-to-volume properties are less than the other four drugs. It's important to note, though, that the circular polymer structure of 2HPβCD limits the amount of surface area that can expand.

#### Water penetration

The CD cavity in an aqueous solution is usually occupied by water molecules in the absence of a guest molecule. X-ray and neutron diffraction studies have confirmed that the cavity of α-CD, β-CD, and γ-CD contains an average of 2.6, 6.5, and 8.8 water molecules, respectively, distributed across multiple sites within the cavity^69–74^. These confined waters are unable to complete their hydrogen bond network as they would in a bulk medium, which creates a high-energy state. When a ligand enters the cavity, these high-energy waters are released, making the cavity-drug complexation energetically favorable^75–78^. Consequently, the number of water molecules in various layers was determined by considering spheres of various radii in the center of the 2HPβCD cavity, as seen in Table 2. Our results show that the number of water molecules in the innermost layer (0–0.5 nm) and the outermost layer (0.9–1.0 nm) is significantly reduced in 2HPβCD compared to βCD^79^, which is one of the most noticeable changes caused by functionalizing the native βCD. The total water accumulation within a 1 nm radius from the center of 2HPβCD is approximately 19% lower than that of βCD, but the number of water molecules in other layers is similar to βCD^27^.

**Table 2:** Number of water molecules in different spheres inside the 2HPβCD cavity.

Systems	Distance				
	0–0.5 nm	0.5–0.8 nm	0.8–0.9 nm	0.9–1.0 nm	0–1.0 nm (total)
Water + 2HβCD (reference)	1.57 (± 0.06)	21.59 (± 0.07)	23.01 (± 0.10)	34.70 (± 0.14)	80.87 (± 0.17)
Water + 2HβCD + DZM	0.06 (± 0.01)	12.50 (± 0.09)	19.48 (± 0.11)	36.55 (± 0.12)	68.59 (± 0.15)
Water + 2HβCD + NDM	0.86 (± 0.08)	9.14 (± 0.08)	18.60 (± 0.14)	38.47 (± 0.18)	67.07 (± 0.24)
Water + 2HβCD + CDP	0.33 (± 0.01)	12.09 (± 0.10)	18.08 (± 0.12)	35.18 (± 0.15)	65.68 (± 0.17)
Water + 2HβCD + NZP	0.20 (± 0.01)	12.10 (± 0.11)	20.78 (± 0.21)	34.19 (± 0.20)	67.27 (± 0.16)
Water + 2HβCD + CLZ	0.33 (± 0.01)	12.21 (± 0.12)	19.36 (± 0.13)	35.64 (± 0.19)	67.54 (± 0.22)

The loading of BZDs into 2HPβCD cavities resulted in significant water outflow in the three inner layers (0.0–0.5, 0.5–0.8, and 0.8–0.9 nm), with the effect being especially noticeable in the first two inner layers. In the presence of NDM, however, water outflow from the innermost layer (0.0–0.5 nm) was significantly lower than in other systems because of incomplete loading (as depicted in Fig. 2). The first layer's (0.0–0.5 nm) largest water outflow is brought on by DZM (0.06), followed by NZP (0.2), and then CDP (0.33) and CLZ (0.33). In contrast to the NDM system, in the presence of other BZDs, the water outflow in the second layer (0.5–0.8 nm) is nearly identical. All systems experience a similar change in the number of water molecules within the third layer (0.8–0.9 nm). However, the presence of NZP oxygen atoms at the end of the PHR and their ability to effectively attract water molecules results in a slightly higher number of water molecules (20.78) within this layer compared to other systems. Once again, the observation that the drug caused water outflow from the center of 2HPβCD is confirmed by examining the radial distribution function (RDF), Fig. S4. Compared to βCD, there was significantly less water accumulation in the center of 2HPβCD^80^. As a result, the first peak in 2HPβCD is considerably smaller than that of βCD.

### Thermodynamic properties

The interaction energies between solute and solvent play a crucial role in determining the solubility and stability of a compound in a solution. These energies are primarily governed by Coulombic, vdW, and hydrogen bonding interactions. The strength and directionality of these interactions depend on the chemical nature and geometry of both the solute and solvent molecules. Understanding these interactions is essential for designing new drug molecules, optimizing reaction conditions, and developing new materials^56,81^.

#### Analysis of energies

**Figure 6:**
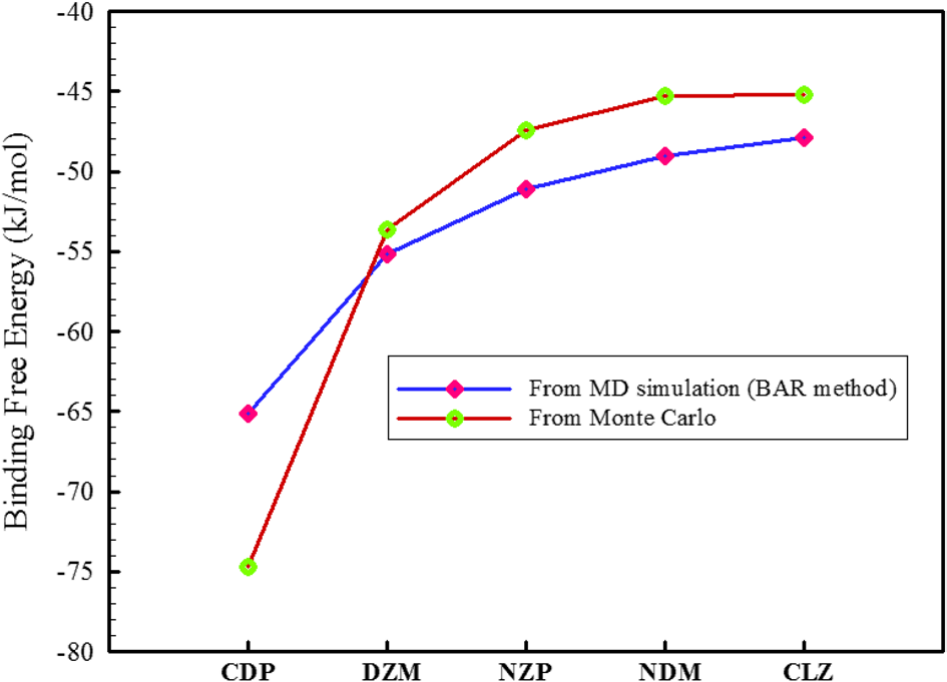
Binding free energies of different BZDs to 2HPβCD.

The binding free energy (ΔG_bind_) between a drug and its carrier is a critical factor in drug delivery efficiency and stability, as it determines the strength of the complex formed and the release rate of the drug from the CD cavity, and can be influenced by various factors, including the size and shape of the drug molecule and the type of CD used^31,82^. As previously mentioned, we used the BAR and MC techniques to determine the binding free energies. Figure 6 depicts that all BZDs have a negative ΔG_bind_ which means that the binding process is spontaneous and thermodynamically favorable. This binding interaction also improves the solubility of the BZDs. The thermodynamic order for ΔG_bind_ of BZDs was consistent in both methods. Both methods showed that CDP and DZM had the highest affinity for binding, despite being predicted by different methods.

Using established and more accurate density functional theory can provide us with a more detailed understanding of drugs. The energies of the drug compounds’ frontier orbitals are listed in Table S1, alongside their HOMO–LUMO energy gaps. A compound’s activity is related to its HOMO–LUMO energy gap, which represents its ability to transfer electrons. The greater the HOMO–LUMO gap of a substance, the less chemically reactive it is^83^. The studied compounds follow the order of CLZ > NDM > NZP > DZM > CDP in terms of ΔE_gap_. In terms of ΔE_gap_, the studied compounds are in the following order: CLZ > NDM > NZP > DZM > CDP. We also calculated the chemical hardness (ƞ), which indicates a compound’s chemical behavior and resistance to polarization (Table S1). The order of CLZ > NDM > NZP > DZM > CDP is maintained for ƞ as well. A drug’s binding free energy to 2HPβCD is affected by changes in ΔE_gap_ and ƞ. A drug with higher chemical activity and less hardness is more likely to bind to 2HPβCD, thermodynamically. The electrophilicity index (ω), a measure of compound’s ability to receive electrons, is also provided in Table S1. DZM and CDP have higher ω values, indicating that they are more electrophilic. The presence of oxygen atoms with free electron pairs in the 2HPβCD molecule may play a role in the interaction of CDP and DZM with 2HPβCD, causing a greater tendency to interaction. It is important to note that the binding free energy is influenced by a variety of molecular forces that occur between the drug and receptor, including Coulombics, vdW, hydrogen bonding, and hydrophobic interactions. Hence, we examined the interaction energies between various components in simulated systems, and the results are summarized in Table 3. Our results revealed that vdW interactions are the primary contributor to binding a BZD to 2HPβCD in all the systems. The Coulombic interactions accounted for only 7–16% of the total interaction energies, with the highest and lowest percentages observed in NZP/2HPβCD (15.7%) and NDM/2HPβCD (7.6%), respectively. While the vdW interaction of DZM/2HPβCD was the strongest at − 153.23 kJ/mol, the results suggest that the interaction of CDP/2HPβCD is the most favorable thermodynamically, with a sum of vdW and Coulombic energies of − 171.3 kJ/mol. Once BZDs interact with water in systems without 2HPβCD, both vdW and Coulombic have a high attraction. Thus, it is essential to consider both factors, as they might significantly affect the drug's solubility.

**Table 3:** Analyzing of energies in different simulated systems.

System	Interaction energy (kJ/mol)					
	Coulombic between water and drug	vdW between water and drug	Coulombic between drug and 2HPβCD	vdW between drug and 2HPβCD	Coulombic between water and 2HPβCD	vdW between water and 2HPβCD
Water + 2HPβCD + DZM	− 38.03 (± 0.88)	− 31.14 (± 0.15)	− 15.94 (± 0.79)	− 153.23 (± 0.65)	− 271.52 (± 2.50)	− 333.46 (± 0.39)
Water + 2HPβCD + NDM	− 71.67 (± 0.39)	− 30.07 (± 0.62)	− 9.76 (± 0.60)	− 117.30 (± 1.50)	− 271.10 (± 2.40)	− 323.47 (± 1.80)
Water + 2HPβCD + CDP	− 57.45 (± 0.64)	− 32.96 (± 0.12)	− 19.52 (± 0.56)	− 151.78 (± 0.68)	− 259.92 (± 1.10)	− 331.18 (± 0.72)
Water + 2HPβCD + NZP	− 76.17 (± 5.90)	− 30.49 (± 2.10)	− 26.12 (± 3.10)	− 139.98 (± 1.70)	− 255.90 (± 1.20)	− 331.38 (± 1.20)
Water + 2HPβCD + CLZ	− 79.32 (± 3.3)	− 30.00 (± 0.45)	− 16.61 (± 1.3)	− 149.28 (± 3.00)	− 268.42 (± 1.7)	− 333.66 (± 0.72)
Water + DZM	− 75.63 (± 0.08)	− 104.60 (± 0.12)	–	–	–	–
Water + NDM	− 97.62 (± 0.23)	− 90.08 (± 0.08)	–	–	–	–
Water + CDP	− 100.48 (± 0.27)	− 104.72 (± 0.22)	–	–	–	–
Water + NZP	− 125.63 (± 0.13)	− 95.06 (± 0.11)	–	–	–	–
Water + CLZ	− 124.21 (± 0.43)	− 98.56 (± 0.29)	–	–	–	–
Water + 2HPβCD	–	–	–	–	310.85 (± 4.20)	− 354.98 (± 3.20)

In the case of DZM and CDP, vdW contribution in the interaction energy with water molecules is greater (~ − 104 kJ/mol) than Coulombic. While for other drugs, Coulombic interactions are the main contributor to their dissolution. However, when 2HPβCD is present, the dominant energy in all systems is Coulombic, although vdW still plays a significant role and cannot be ignored. The presence of 2HPβCD causes a strong vdW repulsion between water and drugs, reducing their vdW (becoming more positive) by about 66–70%, while Coulombic contributions are reduced by 26–50%. Examining the interaction energy between 2HPβCD and water reveals that both Coulombic and vdW energies have a significant contribution in the absence of BZDs, but their contributions became less in the presence of BZDs. The reduction is more noticeable for Coulombic, with a decline of between 13 and 18%, while for vdW decrease of between 7 and 9%.

#### Hydrogen bonds

The stability of the supramolecular complex is directly correlated with a greater deficiency in hydrogen bonds for cavity waters, but this correlation is not substantial. Besides that, host–guest interaction and host-water hydrogen bonds can affect the inclusion complex's stability. Hence, the examination of diverse forms of hydrogen bonds that arise between drugs and 2HPβCD or drugs and water molecules has been considered. In this work, H-bonds are determined by a maximum distance of 3.5 Å between acceptor and hydrogen and also a maximum angle of 30º between acceptor-hydrogen and hydrogen-donor vectors^84^. Table 4 presents the results of different types of hydrogen bonds (along with lifetime) observed in the simulated systems. The first finding concerns the hydrogen bond between water and 2HPβCD is that the average number of hydrogen bonds in the reference system was 20.53, which slightly decreased in the presence of BZDs. However, the quality or lifetime of the hydrogen bond remained stable at around 1.8 picoseconds (ps) in all systems. Out of all the oxygen atoms in 2HPβCD, O3 formed the most hydrogen bonds (Table S2), indicating that it is the most accessible oxygen atom for water. The presence of all drugs except NZP increases the number of hydrogen bonds between O3 and water. This indicates that BZDs can impact the hydrogen bond formation in the system. Additionally, O6 creates several hydrogen bonds with water molecules, which always increase in the presence of BZDs. However, this increase is not very noticeable in the presence of NDM and NZP. On the other hand, O1 and O2 form fewer hydrogen bonds with water when BZDs are present, and O1 hydrogen bonds decrease more significantly than those of O2.

**Table 4:** Average number of different hydrogen bonds in the simulated systems.

System	H-bond			
	Between water and drug (lifetime)	Between water and 2HPβCD (lifetime)	Between drug and 2HPβCD (lifetime)	Between 2HPβCD and 2HPβCD (lifetime)
Water + 2HPβCD (reference)	–	20.53 (1.80)	–	4.19 (2.72)
Water + 2HPβCD + DZM	1.04 (4.30)	18.05 (1.82)	0.27 (2.01)	6.04 (3.87)
Water + DZM	2.15 (1.83)	–	–	–
Water + 2HPβCD + NDM	2.25 (4.14)	17.65 (1.80)	0.17 (1.54)	6.18 (3.56)
Water + NDM	2.76 (2.77)	–	–	–
Water + 2HPβCD + CDP	1.64 (3.90)	16.85 (1.77)	0.31 (1.44)	7.27 (4.10)
Water + CDP	2.90 (2.52)	–	–	–
Water + 2HPβCD + NZP	2.54 (2.86)	17.00 (1.73)	1.00 (2.74)	5.30 (3.50)
Water + NZP	3.81 (2.13)	–	–	–
Water + 2HPβCD + CLZ	2.52 (2.96)	17.62 (1.79)	0.76 (1.94)	6.48 (3.83)
Water + CLZ	3.74 (2.26)	–	–	–

In the presence of BZDs, O1 and O2 seem to have an adverse effect on the dissolution of 2HPβCD, whereas O3 and O6 have a positive effect. When compared to other BZDs, CLZ and NZP generate a higher number of hydrogen bonds with water, regardless of the presence of 2HPβCD. As a result of having more oxygen atoms in their molecular structure than other BZDs (Fig. 1), CLZ and NZP formed the most hydrogen bonds with water. Consistent with the binding free energies indicating that CDP and DZM have the greatest tendency to load inside the 2HPβCD, they also exhibit the greatest reduction in the number of hydrogen bonds with water in this situation, with 1.64 and 1.04 values, respectively. In addition to the favorable and potent vdW interactions, it appears that the high hydrogen bond reduction of these two drugs with water also promotes binding. It is crucial to note that while the number of hydrogen bonds between DZM and CDP and water has decreased, the strength of these bonds has greatly improved. Consequently, their lifetimes have increased from 1.83 and 2.52 ps to 4.3 and 3.9 ps, respectively, in the presence of 2HPβCD. The average number of hydrogen bonds formed between 2HPβCD/2HPβCD while BZDs are present are more than the reference system (4.19). All BZDs trigger an increase in the 2HPβCD/2HPβCD hydrogen bond, with the largest increase observed when CDP is present, which has a lifetime of 7.27 ps. Although BZDs lead to a decrease in hydrogen bonds between 2HPβCD and water, and an increase in hydrogen bonds between 2HPβCD molecules (which has a negative impact on the solubility of 2HPβCD), they still form substantial hydrogen bonds with water and have strong Coulombic and vdW interactions with water. Therefore, in the presence of BZDs, we could still expect 2HPβCD having a significant solubility in water. Because of the BZDs' molecular structure, 2HPβCD/BZDs can also form hydrogen bonds, although not much. Most hydrogen bonds have been formed between NZP and 2HPβCD. Moreover, O1 and O2 seem to have the greatest propensity to establish hydrogen bonds with NZP (Table S2).

## Conclusion

We studied the interaction between several BZD drugs and the 2HPβCD molecules. Our findings show that, except for NDM, which is takes longer time, the BZD molecules bind quickly to the 2HPβCD cavity. Moreover, NDM enters the cavity partially through the primary hydroxyl rim, while their adjacent rings are on the cavity’s exterior, close to the water. Hydroxypropylation at the O2 position of βCD and formation 2HPβCD alters the balance between areas of primary and secondary hydroxyl rims, leading to a more cylindrical shape and changes in the distances between the rings in 2HPβCD. The presence of BZDs led to an increase in the cavity's volume so that 2HPβCD became more circular. Results showed that BZD-loaded 2HPβCD became more spherical, with an increased surface-to-volume ratio, indicating potential applications in drug delivery. Loading of BZDs into 2HPβCD cavities causes significant water outflow in the inner layers from the center of 2HPβCD. We also determined the binding free energy of BZDs to 2HPβCD (ΔG_bind_), and found that all BZDs have a negative ΔG_bind_, indicating that the binding process is spontaneous and thermodynamically favorable. Our results showed that vdW contribution is dominant in binding the drugs to 2HPβCD, whilst Coulombic interaction accounted for only a small portion of the binding energy. When drugs interact with water in systems without 2HPβCD, both vdW and Coulombic interactions cause high attractive forces, and it is important to consider both factors as they can significantly contribute to the solubility of the drug. In the presence of 2HPβCD, the dominant interaction energy between BZDs/water is the Coulombic energy, although vdW interactions still play a significant role and cannot be ignored. The presence of 2HPβCD causes a strong vdW repulsion between water and drugs, reducing their vdW energy by about 66–70%, while Coulombic contribution are reduced by 26–50%, compare to the system without 2HPβCD. The driving forces for the BZDs binding into the 2HPβCD cavity are the reduction of hydrogen bond numbers between the BZDs and water, strong vdW attractive interaction between them, and strong vdW repulsion with water.

## Supplementary Information

Supplementary Information.

## Data Availability

The datasets used and/or analyzed during the current study available from the corresponding author on reasonable request.
