# Supplementary material for: Insights from molecular dynamics and DFT calculations into the interaction of 1,4-benzodiazepines with 2-hydroxypropyl-βCD in a theoretical study
Source: Sci Rep. 2023 Jun 18;13:9866. doi: 10.1038/s41598-023-36385-w (PMC10277284; doi:10.1038/s41598-023-36385-w)
Supplement: Supplementary file 1 — Supplementary Information. [file 41598_2023_36385_MOESM1_ESM.pdf]

Supplementary information: Insights from Molecular Dynamics and DFT Calculations into the Interaction of 1,4-Benzodiazepines with 2-Hydroxypropyl-βCD in a Theoretical Study

Mokhtar Ganjali Koli[a,b], *Rahime Eshaghi Malekshah*[c,*], *Hossein Hajiabadi*[a]

[a]*InSilicoSci Computational Research Centre, Nikopardazesh Ltd., Karaj, Iran*

[b]*Department of Chemistry, University of Kurdistan, Sanandaj, Iran*

[c]*Department of Chemistry, Faculty of Science, Semnan University, Semnan, Iran*

*** Corresponding Author:**

Email: r.eshaghimalekshah@semnan.ac.ir

Contents

Figure S1: Molecular structure and the numbering in benzodiazepines with 5-phenyl-1H-benzo[e][1,4]diazepin-2(3H)-one (a), and 2-Hydroxypropyl-βCD (b)**…..………………..………………3**

Figure S2: Numbering of BZDs atoms in this study**………………………………………………4**

Figure S3: Root mean square fluctuation of heavy atoms of BZDs**……….…………………...5**

Calculation methods for area and volume of cavity……….….………………………………6

Figure S4: Radial distribution function (RDF) of water around 2HPβCD in different simulated systems**………………………………………………………………………………………7**

Table S1: The universal indices of compounds obtained from DFT-D calculation**……………...8**

Table S2: Average number of different hydrogen bonds between O atoms of 2HPβCD in the simulated systems**…….…………………………………………………………………….…9**

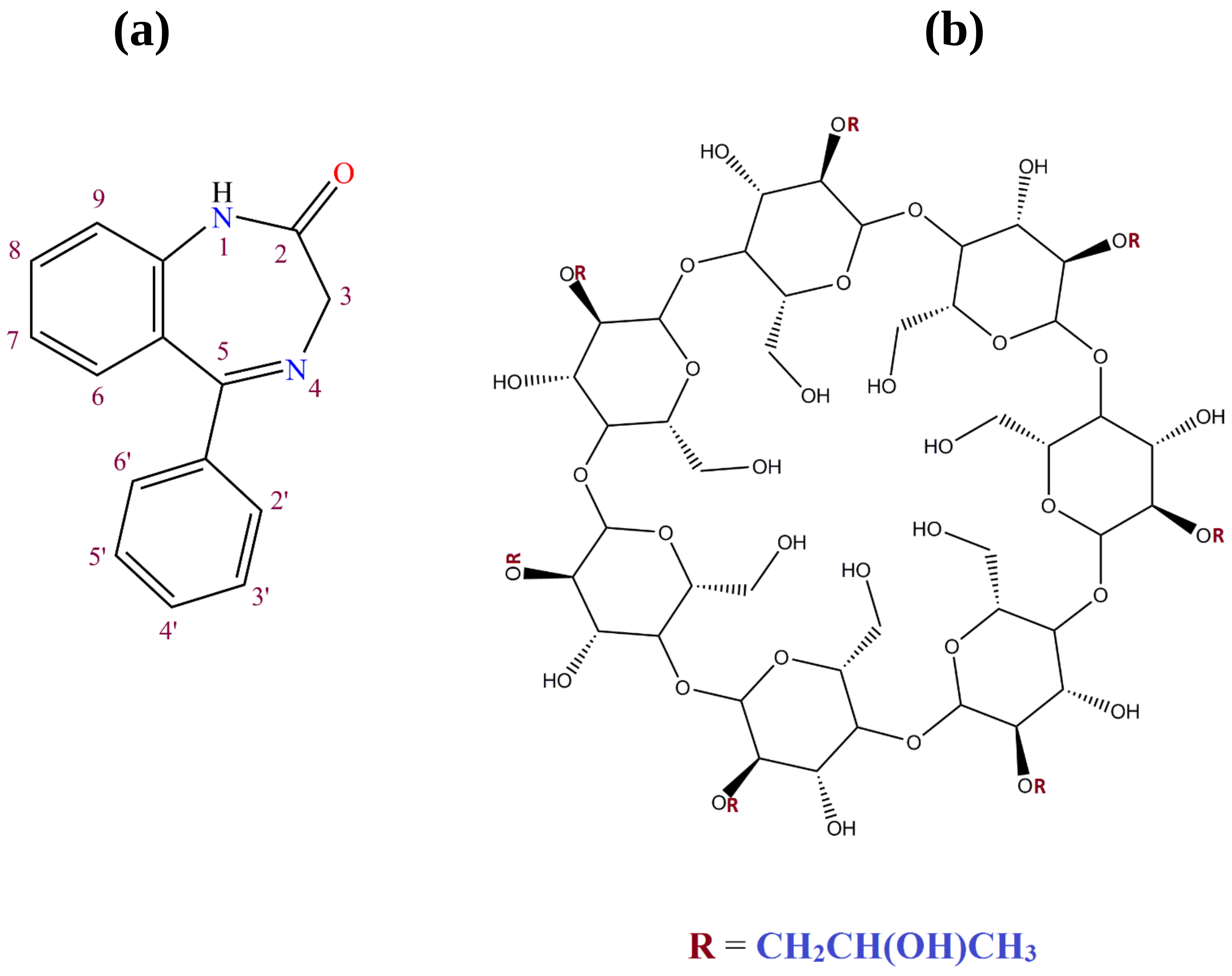

Figure S1: Molecular structure and the numbering in benzodiazepines with 5-phenyl-1H-benzo[e][1,4]diazepin-2(3H)-one (a), and 2-Hydroxypropyl-βCD (b)

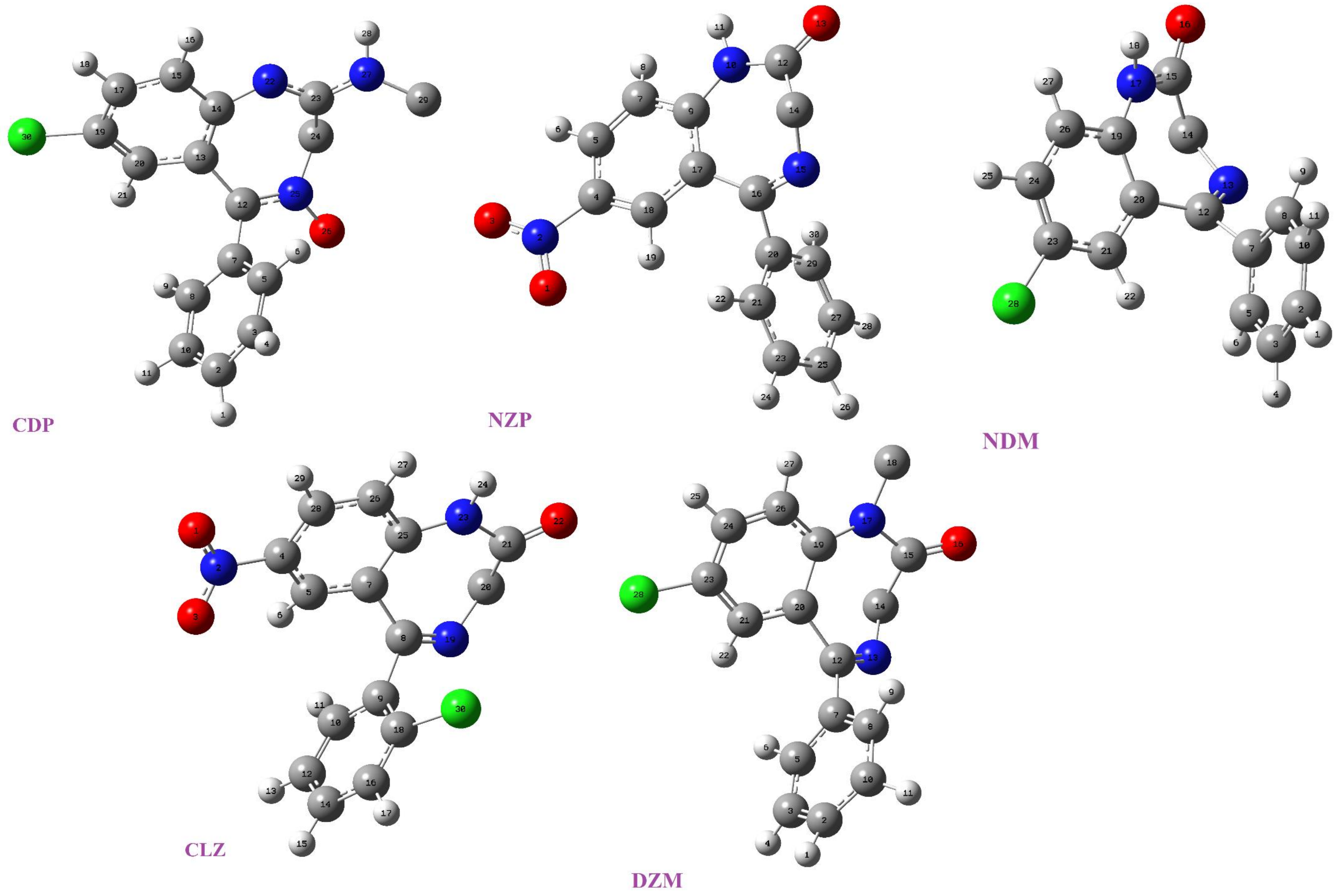

Figure S2: Numbering of BZDs atoms in this study.

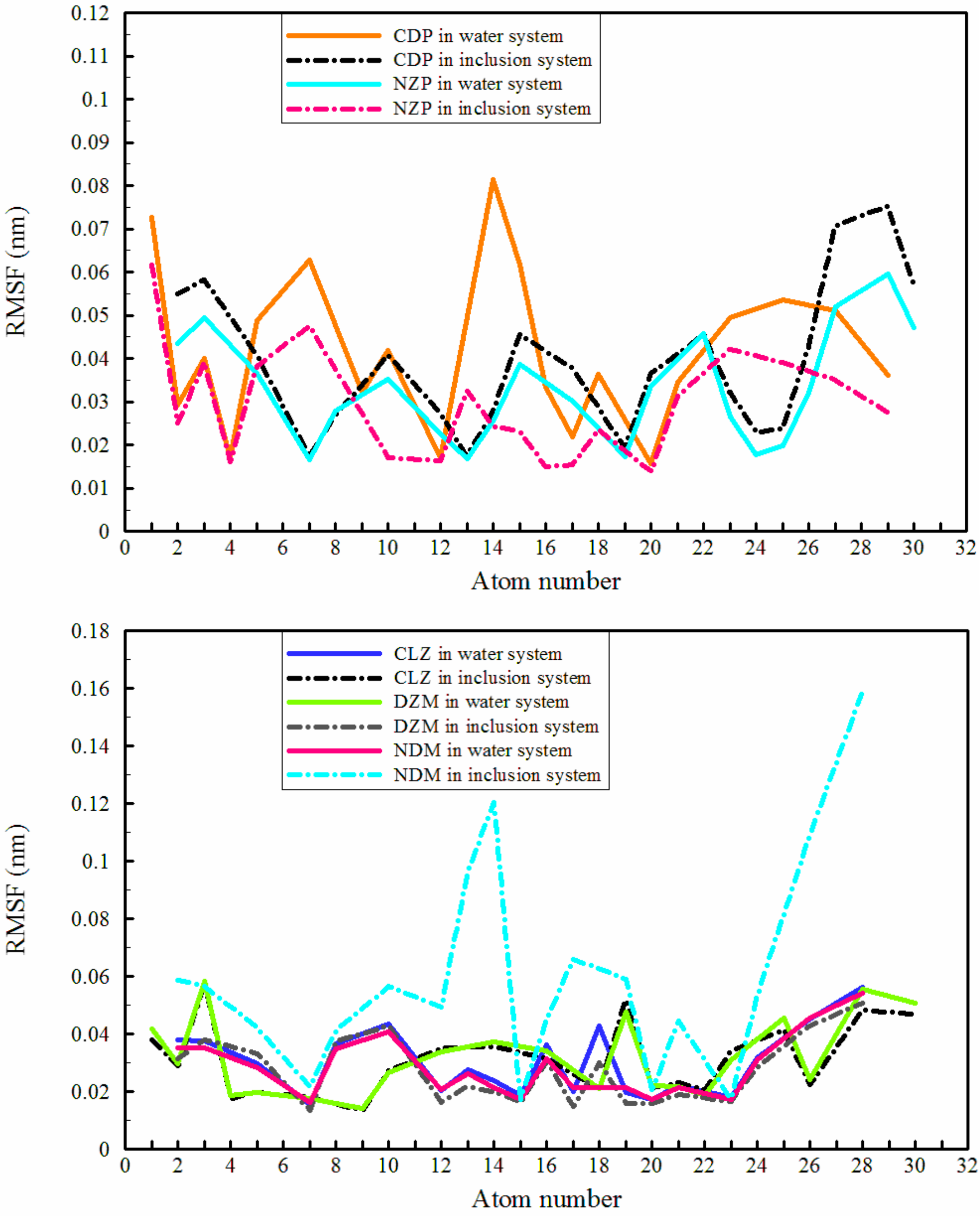

Figure S3: Root mean square fluctuation of all BZDs atoms

Calculation methods for area and volume of cavity

The area of 2HPβCD cavity was calculated by the following equation:

$$A = \frac{\pi}{7}\sum_{i=1}^{7} {r_i}^2$$

Where r_i is the distance between each hydroxyl group and the center of O1 atoms, and the hydroxyl groups at 6- and 3-positions are used for representing the cavity area of primary and secondary hydroxyl rims, respectively.

The CD cavity has a shape that resembles a conical hourglass. As a result, we can approximate its volume by combining the volumes of the truncated cones located at the top and bottom of the cavity, as demonstrated below.

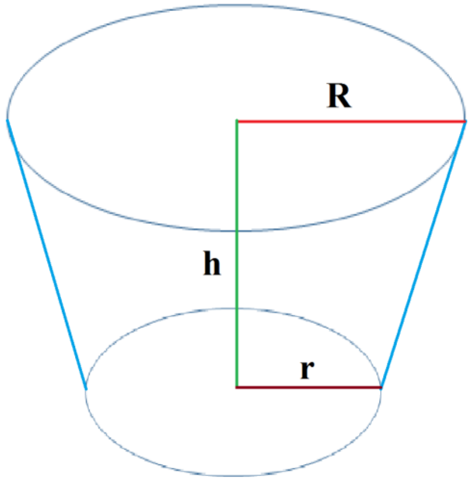

One can determine the volume of a truncated cone with a small radius "r", a large radius "R", and a height "h" by examining its geometry as follows:

$$Vc = \frac{1}{3}\pi h(r^2 + r \times R + R^2)$$

The radius of the O1 rim (Figure 1) was used as the small radius of the cones, while the radius of O2 rim and O6 rim was used as the large radius of the top and the bottom cone, respectively. The height, h, of the cones is sum of h12 and h16 listed in Table 1.

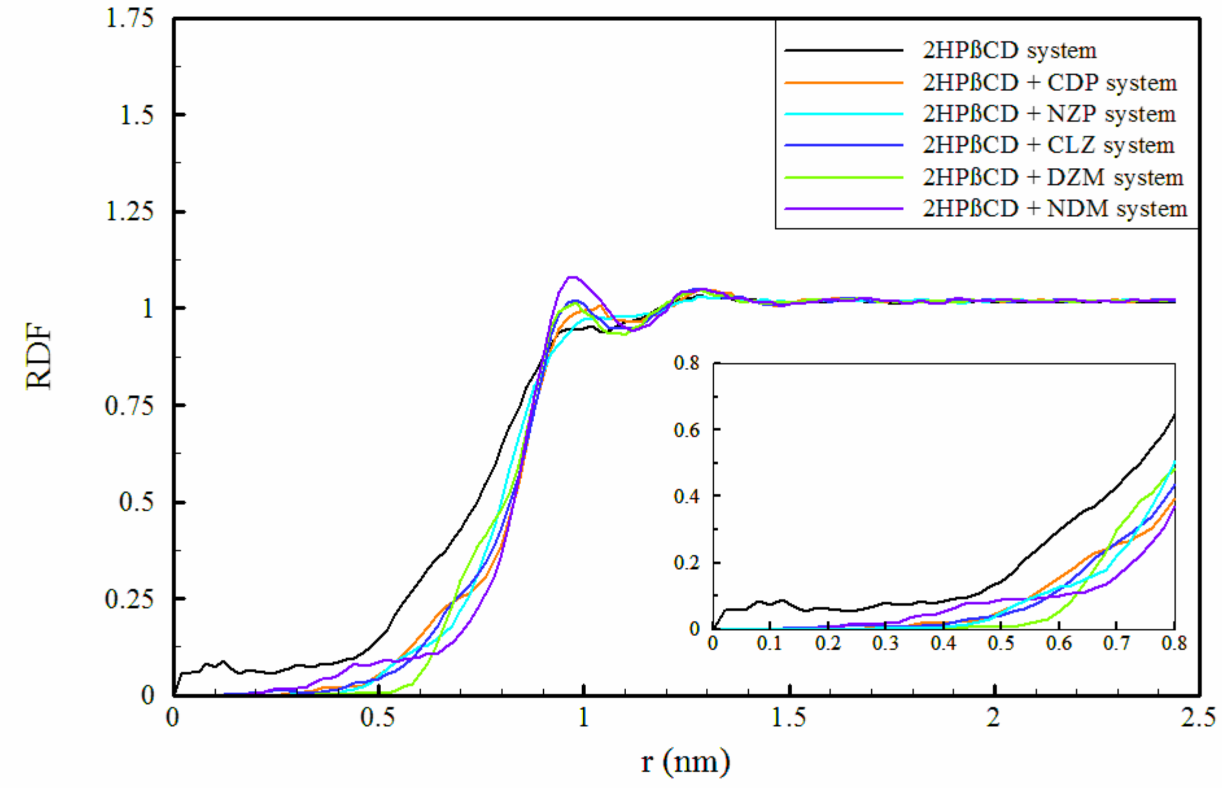

Figure S4: Radial distribution function (RDF) of water around 2HPβCD in different simulated systems.

Table S1: The frontier orbital energy, the HOMO-LUMO energy gap, hardness (η), and electrophilicity index (ω) of the drug compounds.

	**HOMO**	**LUMO**	**E_g**	***η***	***ω***
**CDP**	-6.513	-4.020	2.493	1.246	11.127
**DZM**	-6.527	-4.018	2.509	1.254	11.082
**NZP**	-6.081	-3.108	2.973	1.486	7.101
**NDM**	-6.048	-3.040	3.003	1.504	6.939
**CLZ**	-6.189	-3.152	3.037	1.518	7.217

The universal indices of hardness (η), and global electrophilicity index (ω) were used. They were determined based on the lowest unoccupied molecular orbital (E_{LUMO}), the energy of the highest occupied molecular orbital (E_{HOMO}), energy gap (E_{gap}) for 2-HP- β-CD and drugs which can be calculated using.

$$E_g = E_{HOMO} - E_{LUMO} \quad (1)$$

$$\mu = ½ (E_{HOMO} + E_{LUMO}) \quad (2)$$

$$\eta = ½ (E_{LUMO} - E_{HOMO}) \quad (3)$$

$$\omega = \mu^2/2\eta \quad (4)$$

The energy gap is an important index in the stability of molecules, the higher the energy gap, the more stable the molecule.

Table S2: Average number of different hydrogen bonds between O atoms of 2HPβCD in the simulated systems.

H-bond / System	Between water and O1	Between water and O2	Between water and O3	Between water and O6	Between drug and O1	Between drug and O2	Between drug and O3	Between drug and O6
**Water + 2HPβCD**	1.20 (2.77)	1.71 (2.18)	3.07 (2.12)	1.82 (1.33)	-----	-----	-----	-----
**Water + 2HPβCD + DZM**	0.10 (1.84)	0.78 (1.83)	3.54 (2.87)	2.172 (1.41)	0.00	0.00	0.00	0.00
**Water + 2HPβCD + NDM**	0.35 (2.17)	0.83 (1.83)	3.121 (2.65)	1.83 (1.34)	0.00	0.00	0.00	0.01 (1.55)
**Water + 2HPβCD + CDP**	0.12 (1.94)	0.64 (1.98)	3.10 (2.93)	2.27 (1.41)	0.04 (2.38)	0.08 (1.35)	0.10 (1.38)	0.00
**Water + 2HPβCD + NZP**	0.24 (2.00)	0.72 (2.01)	2.64 (2.50)	1.89 (1.31)	0.238 (6.47)	0.20 (3.24)	0.06 (3.71)	0.001 (1.00)
**Water + 2HPβCD + CLZ**	0.19 (1.95)	0.76 (1.87)	3.28 (2.69)	2.11 (1.41)	0.00	0.070 (1.91)	0.03 (1.35)	0.00
